# Supplementary material for: Deciphering Bacterial Community Structure, Functional Prediction and Food Safety Assessment in Fermented Fruits Using Next-Generation 16S rRNA Amplicon Sequencing
Source: Microorganisms. 2021 Jul 23;9(8):1574. doi: 10.3390/microorganisms9081574 (PMC8401261; doi:10.3390/microorganisms9081574)
Supplement: Supplementary file 1 [file microorganisms-09-01574-s001.zip › microorganisms-1249527-supplementary.pdf]

Supplementary tables

Supplementary Table S1. The PCR conditions for the identification of suspected pathogens

Pathogens	Target gene	Size	Sequence (5′ to 3′)	Reaction Materials Final Volume: 25 ul	PCR Condition	Reference
Acinetobacter baumannii	ITS region	208	p-Ab-ITSF: 5′-CATTATCACGGTAATTAGTG-3′<br>p-AbI-TSB: 5′-AGAGCACTGTGCACTTAAG-3′	DNA: 300 ng<br>Primer: 400 nM<br>Master mix: 5 ul	Pre-denaturation: 94°C 5 min<br>Denaturation: 94°C 30s<br>Annealing: 55°C 30s<br>Extension: 72°C 30s<br>D.A.E. Cycles: 30 cycles<br>Final extension: 72°C 7 min	[1]
Bacillus cereus sensu lato s.l	*Bal*	533	BalF: 5′-TGCAACTGTATTAGCACAAGCT-3′<br>BalR: 5′-TACCACGAAGTTTGTTCACTACT-3′	DNA: 100-300 ng<br>Primer: 400 nM<br>Master mix: 5 ul	Pre-denaturation: 95°C 5 min<br>Denaturation: 94°C 45s<br>Annealing: 55°C 45s<br>Extension: 72°C 45s<br>D.A.E. Cycles: 30 cycles<br>Final extension: 72°C 5 min	[2]
Clostridium difficile	*tcdA*<br>*tcdB*<br>*cdtA*<br>*cdtB*	632,<br>441,<br>260,<br>179	tcdA-F 5′-GTATGGATAGGTGGAGAAGTCAGTG-3′<br>tcdA-R 5′-CGGTCTAGTCCAATAGAGCTAGGTC-3′<br>tcdB-F 5′-GAAGATTTAGGAAATGAAGAAGGTGA-3′<br>tcdB-R 5′-AACCACTATATTCAACTGCTTGTCC-3′<br>cdtA-F 5′-ATGCACAAGACTTACAAAGCTATAGTG-3′<br>cdtA-R 5′-CGAGAATTTGCTTCTATTTGATAATC-3′<br>cdtB-F 5′-ATTGGCAATAATCTATCTCCTGGA-3′<br>cdtB-R 5′-CCAAAATTTCCACTTACTTGTGTTG-3′	DNA: 100 ng<br>Primer: 25 nM tcdA FR,<br>10 nM tcdB FR,<br>200 nM,cdtA FR<br>500 nM,cdtB FR<br>Master mix: 5 ul	Pre-denaturation: 93°C 2 min<br>Denaturation: 93°C 20s<br>Annealing: 60°C 65s<br>Extension: 68°C 70s<br>D.A.E. Cycles: 30 cycles<br>Final extension: 68°C 5 min	[3]

Klebsie pneumoniae	*tyrB*	931	tyrB-F: 5′-GGCTGTACTACAACGATGAC-3′<br>tyrB-R: 3′-TTGAGCAGGTAATCCACTTTG-3′	DNA: 300 ng<br>Primer: 500 nM<br>Master mix: 5 ul	Pre-denaturation: 95°C 5 min<br>Denaturation: 95°C 60s<br>Annealing: 55°C 60s<br>Extension: 72°C 60s<br>D.A.E. Cycles: 35 cycles<br>Final extension: 72°C 10 min	[4]
Staphylococcus aureus	*nuc*	270	nuc-F 5′-GCGATTGATGGTGATACGGTT-3′<br>nuc-R 5′-AGCCAAGCCTTGACGAACTAAAGC-3′	DNA: 300 ng<br>Primer: 400 nM<br>Master mix: 5 ul	Pre-denaturation: 95°C 5 min<br>Denaturation: 94°C 60s<br>Annealing: 55°C 60s<br>Extension: 72°C 60s<br>D.A.E. Cycles: 30 cycles<br>Final extension: 72°C 10 min	[5]
Pseudomonas aeru-ginosa	16S rRNA	956	PA-SS-F: 5′-GGGGGATCTTCGGACCTCA-3′<br>PA-SS-R: 5′-TCCTTAGAGTGCCCACCCG-3′	DNA: 300 ng<br>Primer: 400 nM<br>Master mix: 5 ul	Pre-denaturation: 95°C 2 min<br>Denaturation: 94°C 20s<br>Annealing: 58°C 20s<br>Extension: 72°C 40s<br>D.A.E. Cycles: 25 cycles<br>Final extension: 72°C 4 min	[6]

Streptococcus pneumoniae	*pneumolysin*	75	F 5′-AGCGATAGCTTTCTCCAAGTGG-3′<br>R 5′-CTTAGCCAACAAATCGTTTACCG-3′	DNA: 300 ng<br>Primer: 400 nM<br>Master mix: 5 ul	Pre-denaturation: 94°C 5 min<br>Denaturation: 94°C 45s<br>Annealing: 50°C 90s<br>Extension: 72°C 90s<br>D.A.E. Cycles: 35 cycles<br>Final extension: 72°C 10 min	[7]
nontuberculous mycobacteria, NTM	16s rRNA<br>23S rRNA		Real-Q MTB & NTM Kit	DNA: 300 ng<br>Primer: 400 nM<br>Master mix: 5 ul	Pre-denaturation: 95°C 10 min<br>Denaturation: 95°C 15s<br>Annealing & Extension: 67°C 45s<br>D.A.E. Cycles: 45 cycles	[8]
Stenotrophomonas maltophilia	23S rRNA	278	F 5'GCTGGATTGGTTCTAGGAAAACGC3'<br>R 5'ACGCAGTCACTCCTTGCG3'	DNA: 300 ng<br>Primer: 400 nM<br>Master mix: 5 ul	Pre-denaturation: 94°C 5 min<br>Denaturation: 94°C 45s<br>Annealing: 68°C 45s<br>Extension: 72°C 45s<br>D.A.E. Cycles: 35 cycles<br>Final extension: 72°C 10 min	[7]

Gardmerella vaginalis	16S rRNA		F- GV1 5′-TTACTGGTGTATCACTGTAAGG-3′<br>R- GV3 5′-CCGTCACAGGCTGAACAGT-3′	DNA: 300 ng<br>Primer: 400 nM<br>Master mix: 5 ul	Pre-denaturation: 94°C 5 min<br>Denaturation: 94°C 45s<br>Annealing: 55°C 45s<br>Extension: 72°C 45s<br>D.A.E. Cycles: 35 cycles<br>Final extension: 72°C 10 min	[9]
Chryseobacterium in-dologenes	6S-23S rRNA ISR	561	Chry-16S-F 5′-GCATCAGCCATGGCGCGGTG-3′<br>Chry-23S-R 5′-CCTTAACGATTTCTTTCCTA-3′	DNA: 300 ng<br>Primer: 400 nM<br>Master mix: 5 ul	Pre-denaturation: 94°C 5 min<br>Denaturation: 94°C 45s<br>Annealing: 55°C 45s<br>Extension: 72°C 45s<br>D.A.E. Cycles: 35 cycles<br>Final extension: 72°C 10 min	[10]

Supplementary Table S2. Chemical analysis of fermented fruits from the 2nd stage of fermentation and final products

Process	Samples	pH[1]	°Brix[2] (%)	Organic acid[3] (%)	Ethanol[4] (%)	Measurement time
	Ch1_2nd	3.2	24.0	3.8	0.0	1st month
	Ch2_2nd	3.2	23.3	3.6	0.0	
	Ch3_2nd	3.4	22.5	3.4	0.0	
	Ch1_2nd	3.2	23.6	4.1	0.0	2nd month
	Ch2_2nd	3.4	23.2	3.7	0.0	
	Ch3_2nd	3.3	22.2	3.5	0.0	

2nd stage of fermentation	Ch1_2nd	3.3	23.8	3.8	0.0	3rd month
	Ch2_2nd	3.4	23.2	3.6	2.2	
	Ch3_2nd	3.4	22.2	3.4	2.4	
	Ch1_2nd	3.3	23.6	4.3	0.0	4th month
	Ch2_2nd	3.4	23.2	4.1	0.0	
	Ch3_2nd	3.4	22.2	3.8	0.7	
	Ch1_2nd	3.3	23.2	4.4	0.0	5th month
	Ch2_2nd	3.2	23.0	4.0	0.0	
	Ch3_2nd	3.3	22.0	4.0	0.0	
	Ch1_2nd	3.2	23.4	4.3	0.0	6th month
	Ch2_2nd	3.3	23.0	4.0	1.7	
	Ch3_2nd	3.3	22.0	3.9	1.4	
	Ch1_2nd	3.2	23.4	4.3	0.0	7th month
	Ch2_2nd	3.3	23.0	4.1	1.1	
	Ch3_2nd	3.3	22.0	4.0	0.0	
	Ch1_2nd	3.2	22.8	4.5	0.0	8th month
	Ch2_2nd	3.3	23.0	4.0	0.0	
	Ch3_2nd	3.3	22.0	3.9	1.7	
Product	Pr1_F	2.8	10.8	5.8	2.8	0 day
	Pr2_F	2.6	11.0	5.8	2.7	
	Pr3_F	2.7	11.2	5.9	2.7	

[1] The pH of samples is measured using a DO Microelectronic pH-Vision pH meter (model PHB-9901, Ai-On Industrial Corp., Taipei, Taiwan).

[2] °Brix (Brix) was measured using Brix meter (Lotus, China) following the manufacturer protocol. Brix is mainly made up of sugars but also includes other compounds.

[3] Organic acids content was measured using the principle of acid-base neutralization titration of volumetric analysis. In brief, a 2 ml sample was taken in a beaker with the addition of distilled water to 20 ml (10 times dilution) and titrated with 0.1N NaOH standard solution. The initial and end of titration was recoded and finally, the below formula was used to determine the organic acid contents.

The amount of organic acid in fermented food (%) = (S-B) * F * O.A * D.F / Swt *100

S: The sample test consumed the number of 0.1N NaOH mL

B: The number of 0.1N NaOH mL consumed in the blank test

F: The titer of 0.1N NaOH (acceptable range: 0.9~1.1)

O.A: Organic acid (citric acid based: 0.0064)

D.F: Dilution factor

Swt: sample weight (mL)

Additionally, the manufacture company also uses Agilent Hi-Plex H column with UV detection to separate, identify, and estimate organic acids, including oxalic acid, citric acid, tartaric acid, malic acid, succinic acid, formic acid, and fumaric acid, when they need to show the product quality report.

[4] Ethanol contents were measured using ethyl alcohol Meter (Ethyl alcohol meter PAL-33S, Atago Co., LTD.) following the manufacture protocol.

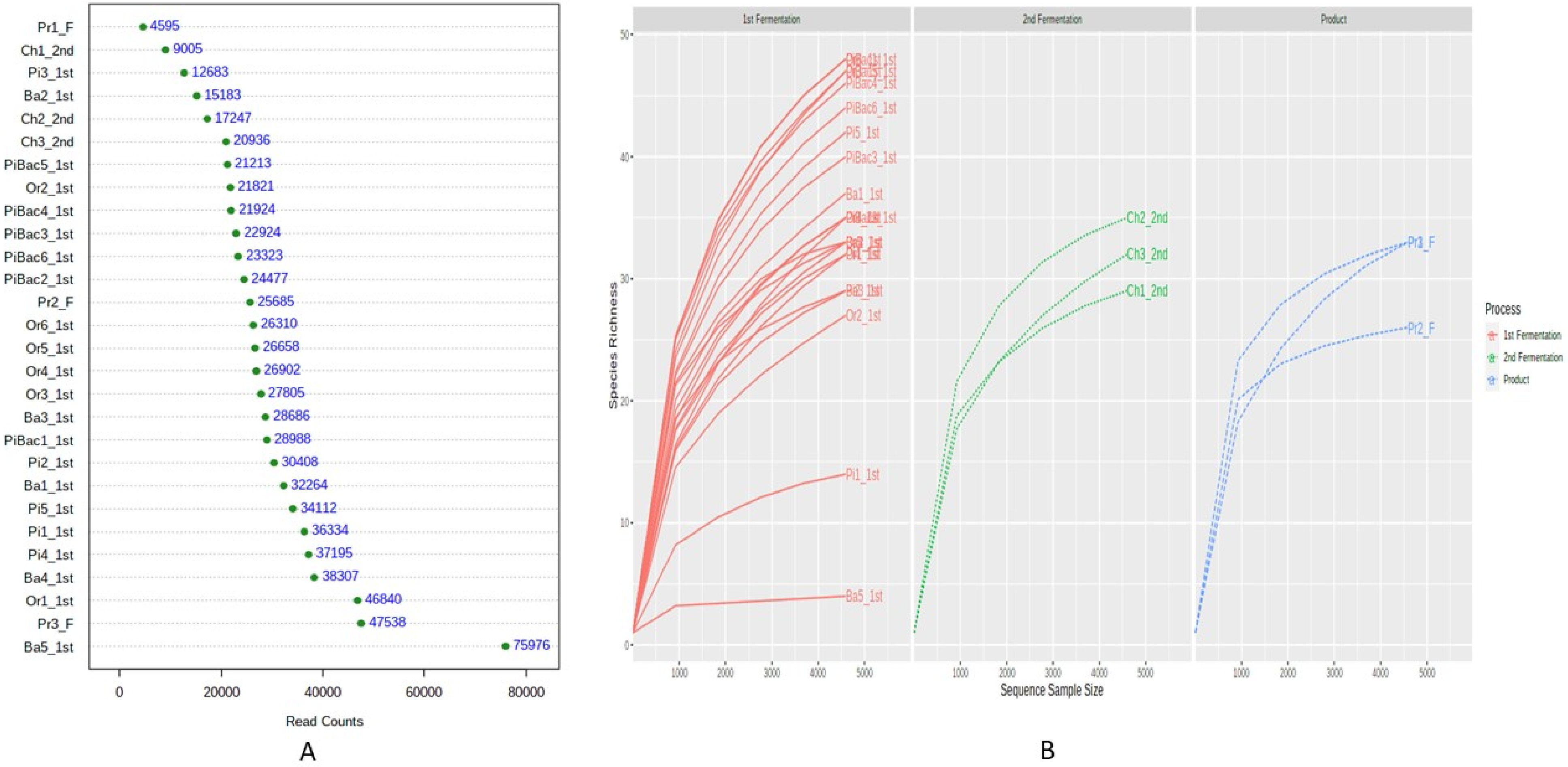

Supplementary Figure S1. Library size overview (A) and alpha rarefaction plot (B) among the fermentation stages and the final products.

Supplementary Table S3. The sequence good's coverage at the minimum sequence depth associated with the fermented samples

samples	no. sequence	goods
Pr1_F	4595	99.89119
Pr2_F	4595	99.93471
Pr3_F	4595	99.8259
Or1_1st	4595	99.8259
Or2_1st	4595	99.76061
Or3_1st	4595	99.6518
Pi-Bac1_1st	4595	99.71708
Pi-Bac2_1st	4595	99.67356
Pi-Bac3_1st	4595	99.76061
Pi1_1st	4595	99.93471
Pi2_1st	4595	99.8259
Ba1_1st	4595	99.69532
Ba2_1st	4595	99.8259
Ba3_1st	4595	99.86942
Ch1_2nd	4595	99.89119
Ch2_2nd	4595	99.86942
Ch3_2nd	4595	99.78237
Or4_1st	4595	99.78237
Or5_1st	4595	99.93471
Or6_1st	4595	99.71708
Pi-Bac4_1st	4595	99.69532

Pi-Bac5_1st	4595	99.6518
Pi-Bac6_1st	4595	99.71708
Pi3_1st	4595	99.78237
Pi4_1st	4595	99.73885
Pi5_1st	4595	99.71708
Ba4_1st	4595	99.73885
Ba5_1st	4595	99.97824

References

1. Chen, T.-L.; Sin, L.-K.; Wu, R.-C.; Shaio, M.-F.; Huang, L.-Y.; Fung, C.-P.; Lee, C.-M.; Cho, W.-L. Comparison of one-tube multiplex PCR, automated ribotyping and intergenic spacer (ITS) sequencing for rapid identification of Acinetobacter baumannii. *Clin Microbiol Infec* **2007**, *13*, 801-806.
2. Das, S.; Surendran, P.; Thampuran, N. PCR-based detection of enterotoxigenic isolates of Bacillus cereus from tropical seafood. *Indian Journal of Medical Research* **2009**, *129*, 316.
3. Chankhamhaengdecha, S.; Hadpanus, P.; Aroonnual, A.; Ngamwongsatit, P.; Chotiprasitsakul, D.; Chongtrakool, P.; Janvilisri, T. Evaluation of multiplex PCR with enhanced spore germination for detection of Clostridium difficile from stool samples of the hospitalized patients. *BioMed research international* **2013**, *2013*.
4. JEong, E.-S.; Lee, K.-S.; HEo, S.-H.; SEo, J.-H.; CHoi, Y.-K. Rapid identification of Klebsiella pneumoniae, Corynebacterium kutscheri, and Streptococcus pneumoniae using triplex polymerase chain reaction in rodents. *Experimental animals* **2013**, *62*, 35-40.
5. Brakstad, O.G.; Aasbakk, K.; Maeland, J.A. Detection of Staphylococcus aureus by polymerase chain reaction amplification of the nuc gene. *Journal of clinical microbiology* **1992**, *30*, 1654-1660.
6. Spilker, T.; Coenye, T.; Vandamme, P.; LiPuma, J.J. PCR-based assay for differentiation of Pseudomonas aeruginosa from other Pseudomonas species recovered from cystic fibrosis patients. *Journal of clinical microbiology* **2004**, *42*, 2074-2079.
7. Greiner, O.; Day, P.J.; Bosshard, P.P.; Imeri, F.; Altwegg, M.; Nadal, D. Quantitative detection of Streptococcus pneumoniae in nasopharyngeal secretions by real-time PCR. *Journal of clinical microbiology* **2001**, *39*, 3129-3134.
8. Kim, Y.N.; Kim, K.M.; Choi, H.N.; Lee, J.H.; Park, H.S.; Jang, K.Y.; Moon, W.S.; Kang, M.J.; Lee, D.G.; Chung, M.J. Clinical usefulness of PCR for differential diagnosis of tuberculosis and nontuberculous mycobacterial infection in paraffin-embedded lung tissues. *The Journal of Molecular Diagnostics*

2015, *17*, 597-604.
9. Zariffard, M.R.; Saifuddin, M.; Sha, B.E.; Spear, G.T. Detection of bacterial vaginosis-related organisms by real-time PCR for Lactobacilli, Gardnerella vaginalis and Mycoplasma hominis. *FEMS Immunology & Medical Microbiology* **2002**, *34*, 277-281.
10. Pridgeon, J.; Klesius, P.; Garcia, J. Identification and virulence of C hryseobacterium indologenes isolated from diseased yellow perch (P erca flavescens). *Journal of applied microbiology* **2013**, *114*, 636-643.
